# Supplementary material for: Boosting DNA vaccine power by lipid nanoparticles surface engineered with amphiphilic bioresorbable copolymer
Source: Mol Ther Nucleic Acids. 2024 Jun 17;35(3):102261. doi: 10.1016/j.omtn.2024.102261 (PMC11278320; doi:10.1016/j.omtn.2024.102261)
Supplement: Document S1. Supplemental methods and Figures S1–S7 [file mmc1.pdf]

OMTN, Volume *35*

Supplemental information

Boosting DNA vaccine power by lipid nanoparticles surface engineered with amphiphilic bioresorbable copolymer

Chung-Hsiang Yang, Kuan-Yin Shen, Hui-Min Ho, Chiung-Yi Huang, Yu-Jhen Cheng, Chih-Chun Pu, Fang-Feng Chiu, Wan-Chun Huang, Hung-Chun Liao, Hsin-Wei Chen, Ching-Len Liao, Shih-Jen Liu, and Ming-Hsi Huang

Supplemental methods

***In vitro* imaging.** After 72-hr transfection with GFP DNA-LNPs, the transfected cells were cultured in 24-well plates (Cat. #142475, Nunc, Thermo-Fisher Scientific, China) in 1 mL culture medium, and images were taken with a fluorescence microscope (Olympus IX73 inverted microscope, Tokyo, Japan). Cell nuclei were stained with Hoechst 33342 dye (Thermo-Fisher Scientific, Rockford, IL, USA) before imaging.

***In vitro* luminescence.** After 72-hr transfection with CBGr99 DNA-LNPs, the transfected cells were harvested and washed with PBS and then lysed using 150 μL of Passive Lysis Buffer (Cat. #E1941, Promega, WI, USA). The luciferase activity was measured by mixing 50 μL of the cell lysate with 50 μL of luciferase substrate (Cat. #E151A, Promega, WI, USA) in a 96-well white plate (Cat. #236108, Nunc, Thermo-Fisher Scientific, Denmark) and then measuring the luminescence by an Orion L Microplate Luminometer (Berthold Detection System, TN, USA). Luciferase activities were expressed as relative light units per second (RLU/s).

RNA extraction and RT-qPCR. RNA was extracted at 24, 48, 72 hrs post-transfection using the RNeasy® Mini Kit (Cat. #74104, QIAGEN, Hilden, Germany) following the manufacturer's protocol. Prior to RNA extraction, the cell culture medium was removed, and the cells were washed once with PBS before being lysed. From the lysates (600 ng of RNA), cDNA was synthesized using SuperScript III Reverse Transcriptase (Cat. #18080044, Thermo-Fisher Scientific, MA, USA) with oligo(dT)12-18 primer (Cat. #18418012, Thermo-Fisher Scientific, MA, USA) following the manufacturer's instructions. Before qPCR reaction, cDNA samples were diluted 3 times in RNase- and DNase-free water. 1 μL cDNA was used in each reaction with 200 nM forwards and reverse primers (Integrated DNA Technologies, Singapore). PCR was performed

with KAPA SYBR® FAST qPCR Master Mix (Cat. #kk4609, KAPA BIOSYSTEMS, MA, USA) on a LightCycler® 480 (Roche Diagnostics, IN, USA) at a total volume of 10 μL in 384-well plates (Roche Diagnostics, IN, USA). The cycling conditions were set as pre-incubation at 95°C for 3 min, followed by 40 cycles of 95°C for 10 sec, 60°C for 20 sec, and 72°C for 1 sec. The program ended with melting curve analysis to check primer dimers. In the experiment, two replicates of cDNA samples were performed along with no transcriptase control or no template control to check genomic DNA contamination. Expression data were analyzed using the Delta-Delta Ct method. The expression of GAPDH was used as a reference control.

DNA-LNP stability. Stability monitoring of the DNA-LNP formulations was performed at 4, 15, 25, and 37°C. At week 2 and week 4, three specimens were withdrawn and tested for their particle size distribution, encapsulation efficiency and transfection of DNA reporter genes in HEK293 cells (human embryonic kidney 293 cells, Cat. #BCRC60019).

***In vivo* luminescence in mice.** BALB/c mice were obtained from the NLABRC (Taipei, Taiwan) and housed at the Animal Center of the NHRI and maintained in accordance with institutional animal care protocols (Protocol No: NHRI-IACUC-109077-A). Transfection of DNA molecules and expression of luciferase protein in mice were measured by using *in vivo* luminescence. Mice were inoculated with predetermined amounts of the DNA-LNP (10 μg per mouse) via the intramuscular (i.m.) route. On Days 1, 2, 3, 4, 7, 10, 14, 21, and 28 after administration, mice were injected intraperitoneally (i.p.) with 100 μL D-luciferin (Cat. # ab143655, Abcam, Cambridge, UK) at a concentration of 15 mg/mL in PBS, and the reaction were performed for 11 minutes. Luminescence signals were collected by an IVIS Spectrum instrument (Perkin Elmer, Waltham, MA, USA), and the luminescence signals in regions of interest (ROIs) were quantified using the imaging software "Living Image".

Supplemental Figures

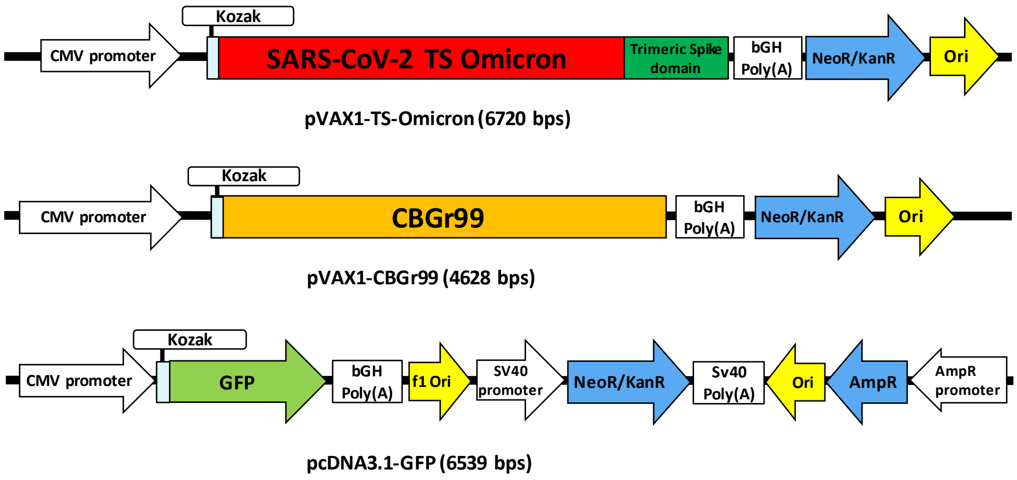

Acronym	Encapsulation efficiency (EE%)	Particle size and electrophoretic mobility		
		Diameter (Z-avg, nm)	Polydispersity index (PDI)	Zeta potential (mV)
TS DNA-LNP	91.4 ± 0.3	127.8 ± 0.4	0.135 ± 0.027	-12.5 ± 1.0
CBGr99 DNA-LNP	93.1 ± 0.2	118.4 ± 0.4	0.118 ± 0.022	-11.2 ± 1.3
GFP DNA-LNP	82.5 ± 0.4	139.2 ± 1.4	0.126 ± 0.011	-24.5 ± 0.4

Figure S1. Plasmid illustration of plasmids used in the present study and fundamental characterization of DNA-LNPs

DNA-LNPs were characterized in terms of encapsulation efficiency (EE%), Z-avg particle size (nm), polydispersity index (PDI), and zeta potential (mV). The results are represented as the mean ± STD of three samples. The molar ratio of lipids was cholesterol:DSPC:SM-102:PLA-PEG of 38.5:10:50:1.5.

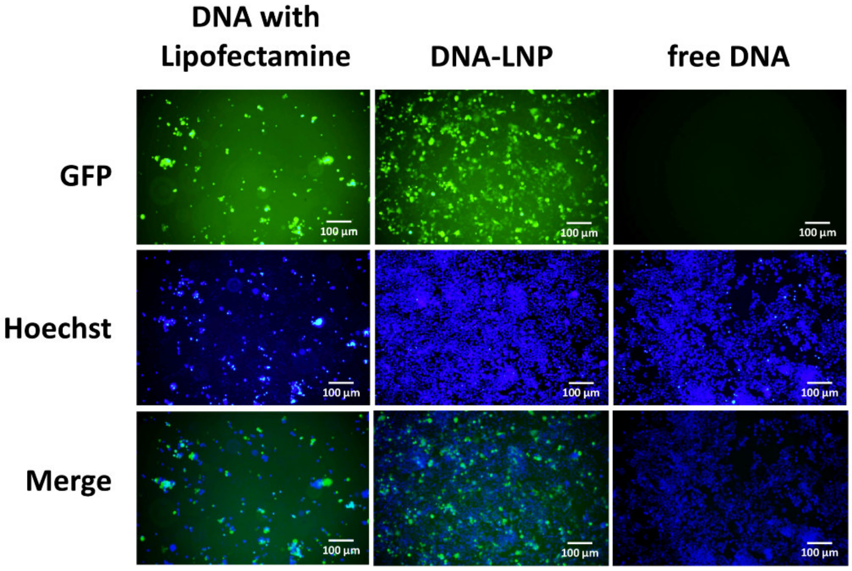

Figure S2. Effects of LNP on DNA transfection in the cell culture system

1 × 10^5 HEK293 cells were treated with GFP-encoding plasmid DNA, either in culture medium (DNA alone), encapsulated within the LNP formulation or in the presence of the conventional transfection reagent Lipofectamine^{TM} 2000. Complexes were prepared by pre-incubating 1 µg DNA and 3 µL Lipofectamine^{TM} 2000 in 100 µL serum-free DMEM at room temperature for 20 min following manufacturer instructions. Observation of protein expression by fluorescence microscopy (green channel) and Hoechst staining (blue channel) of cell nuclei after 72 hrs. Scale bar: 100 µm. The molar ratio of lipids was cholesterol:DSPC:SM-102:PLA-PEG of 38.5:10:50:1.5.

A

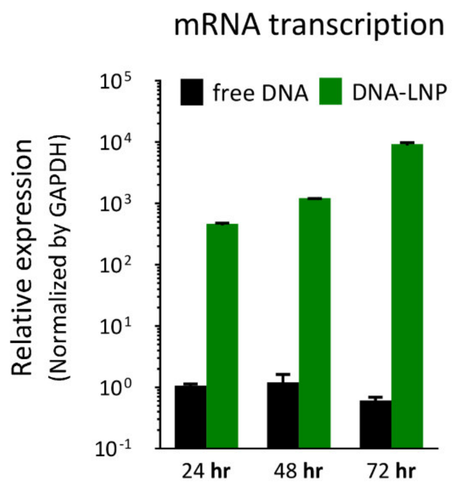

B

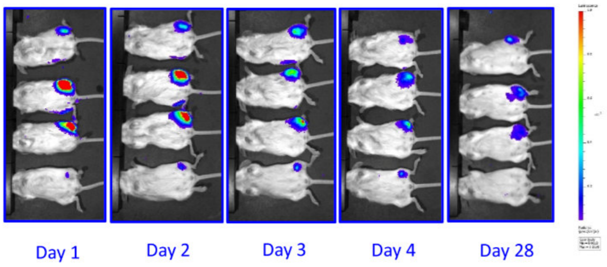

C

Gene	Primer sequence	Length
CBGr99	5'-GTGTCACCGTCTTGGTCTAC-3'<br>5'-CGCGGAGACCGACCATAAAG-3'	64 bps
GAPDH	5'-GAGAAGGCTGGGGCTCATTT-3'<br>5'-TGATGACCCTTTTGGCTCCC-3'	48 bps

Figure S3. Transfection of DNA-LNP formulation *in vitro* and *in vivo*

(A) Transcription and protein expression of the DNA-LNP formulation in the cell culture system. 1×10^5 HEK293 cells were treated with the CBGr99 luciferase plasmid, either in culture medium (free DNA) or in the LNP formulation. The molar ratio of lipids was cholesterol:DSPC:SM-102:PLA-PEG of 38.5:10:50:1.5 (CBGr99 DNA-LNP-F2). Luciferase mRNA quantification in HEK293 cells at 24-, 48-, and 72-hr post transfection was analyzed by the Delta-Delta Ct method. Luciferase activity at 72-hr post transfection was measured as relative light units (RLU/s) in protein lysates from HEK293 cells transfected with plasmid encoding CBGr99 luciferase. (B) Bioluminescence images at the site of injection in a mouse model. Four BALB/c mice were injected intramuscularly with DNA-LNPs (10 µg CBGr99 luciferase plasmid per mouse). Representative IVIS images of DNA-LNPs at selected time points. (C) Primers used for RT-qPCR analysis.

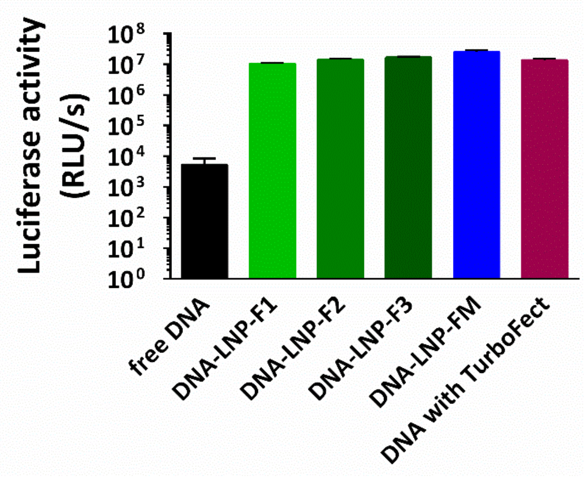

Figure S4. Impact of the lipid contents on the transfection of DNA-LNP formulation in the cell culture system

1×10^5 HEK293 cells were treated with the CBGr99 luciferase plasmid, either in culture medium (DNA alone), within different LNP formulation or in the presence of the conventional transfection reagent Turbofect™. Luciferase activity at 72-hr post transfection was measured as relative light units (RLU/s) in protein lysates from HEK293 cells transfected with plasmid encoding CBGr99 luciferase. DNA-LNP-F1, -F2, -F3, -FM represent DNA-LNPs without PEGylated lipid or with 1.5% PLA-PEG or 3.0% PLA-PEG, or 1.5% DMG-PEG, respectively. Data are represented as the mean ± STD of three replicates of each sample.

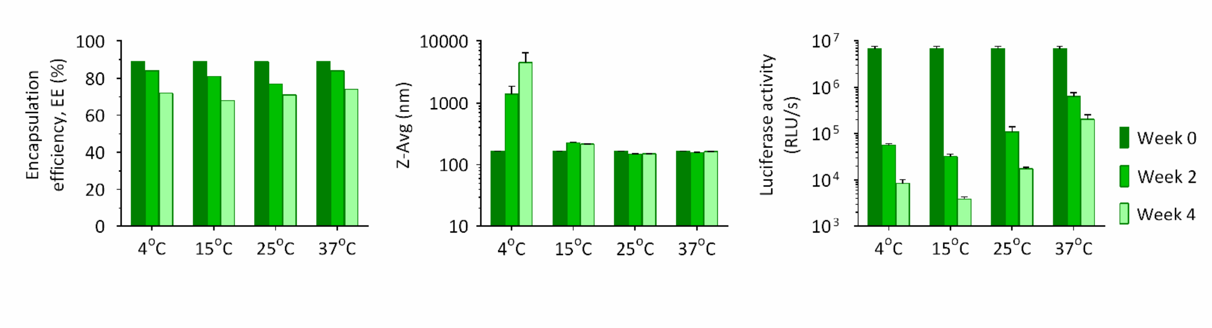

Figure S5. Characteristic changes of DNA-LNP upon storage at 4°C, 15°C, 25°C and 37°C

DNA-LNP was prepared with a standard molar ratio of lipids of cholesterol:DSPC:SM-102:PLA-PEG of 38.5:10:50:1.5 and monitored in terms of encapsulation efficiency (EE%), Z-avg particle size (nm) and luciferase activity in HEK293 cells 72 hrs post transfection, respectively. The results are represented as the mean ± STD of three different samples. The molar ratio of lipids was cholesterol:DSPC:SM-102:PLA-PEG of 38.5:10:50:1.5 (DNA-LNP-F2).

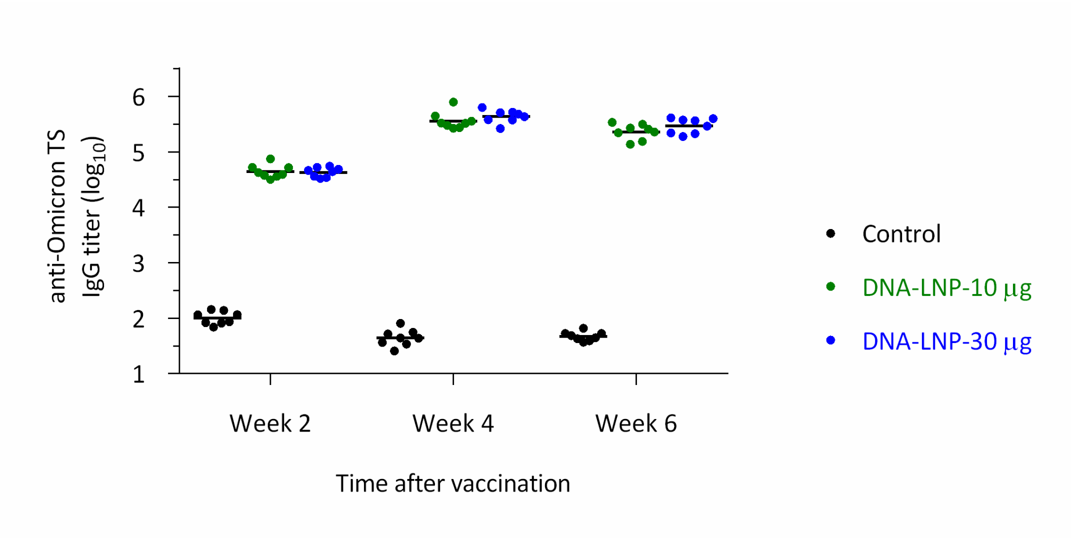

Figure S6. Omicron TS-specific IgG titers in hamsters injected with a two-dose TSomi DNA-LNP vaccination regimen

Hamsters (n = 8) were injected with TSomi DNA-LNP through the i.m. route on Day 0. At week 2, all hamsters were boosted through i.m. injection of the same vaccine formulations. The IgG antibodies in serum against trimeric spike protein are expressed as individual values with the GMT, which were obtained by enzyme-linked immunosorbent assay (ELISA).

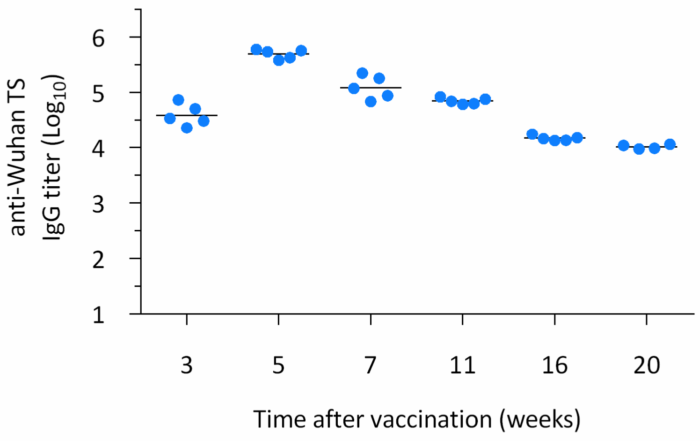

Figure S7. Wuhan TS-specific IgG titers in hamsters injected with a two-dose Wuhan mRNA-LNP vaccination regimen

Hamsters (*n* = 5) injected with 10 μg Wuhan mRNA-LNP through the i.m. injection on Day 0 and Day 21. The IgG antibodies in serum against trimeric spike protein are expressed as individual values with the GMT, which were obtained by enzyme-linked immunosorbent assay (ELISA).
