# Supplementary material for: Boosting DNA vaccine power by lipid nanoparticles surface engineered with amphiphilic bioresorbable copolymer
Source: Mol Ther Nucleic Acids. 2024 Jun 17;35(3):102261. doi: 10.1016/j.omtn.2024.102261 (PMC11278320; doi:10.1016/j.omtn.2024.102261)
Supplement: Document S2. Article plus supplemental information [file mmc2.pdf]

Molecular Therapy
Nucleic Acids
Original Article

Boosting DNA vaccine power by lipid nanoparticles surface engineered with amphiphilic bioresorbable copolymer

Chung-Hsiang Yang,[1,5] Kuan-Yin Shen,[1,5] Hui-Min Ho,[1] Chiung-Yi Huang,[1] Yu-Jhen Cheng,[1] Chih-Chun Pu,[1] Fang-Feng Chiu,[1] Wan-Chun Huang,[1] Hung-Chun Liao,[1] Hsin-Wei Chen,[1,2,3] Ching-Len Liao,[1] Shih-Jen Liu,[1,2,3] and Ming-Hsi Huang[1,2,3,4]

[1]National Institute of Infectious Diseases and Vaccinology, National Health Research Institutes, Miaoli 35053, Taiwan; [2]Graduate Institute of Biomedical Sciences, China Medical University, Taichung 40402, Taiwan; [3]Graduate Institute of Medicine, Kaohsiung Medical University, Kaohsiung 80708, Taiwan; [4]Biotechnology Center, National Chung Hsing University, Taichung 40227, Taiwan

Successful DNA vaccination generally requires the aid of either a viral vector within vaccine components or an electroporation device into the muscle or skin of the host. However, these systems come with certain obstacles, including limited transgene capacity, broad preexisting immunity in humans, and substantial cell death caused by high voltage pulses, respectively. In this study, we repurposed the use of an amphiphilic bioresorbable copolymer (ABC), called PLA-PEG, as a surface engineering agent that conciliates lipid nanoparticles (LNPs) between stability during preparation and biocompatibility post-vaccination. The LNP carrier can be loaded with severe acute respiratory syndrome coronavirus 2 (SARS-CoV-2) spike-specific DNA; in this form, the DNA-LNP is immunogenic in hamsters and elicits protective immunity following DNA-LNP vaccination against heterologous virus challenge or as a hybrid-type vaccine booster against SARS-CoV-2 variants. The data provide comprehensive information on the relationships between LNP composition, manufacturing process, and vaccine efficacy. The outcomes of this study offer new insights into designing next-generation LNP formulations and pave the way for boosting vaccine power to combat existing and possible emerging infectious diseases/pathogens.

INTRODUCTION

Currently, a series of vaccine candidates against emerging viral pandemic COVID-19 are in development worldwide, and several of these candidate vaccines are either authorized for emergency use or approved for use in humans.[1–4] Among these, nucleic acid (NA)-based vaccines contain a section of genetic material derived from severe acute respiratory syndrome coronavirus 2 (SARS-CoV-2) that prompts a person's own cells to generate parts of the targeted virions, and as a result, generate immunity.[1,2] Despite mRNA having received major interest and entering a new era in vaccinology, RNA molecules exhibit a low stability and must be protected from enzymatic digestion to achieve successful targeted delivery.[4] Recently, lipid nanoparticle (LNP) technology was successfully advanced and became more sophisticated, highlighting the gap between the advantages of mRNA carrier platforms and the availability of automated microfluidic manufacturing processes to scale up their production.[4–6] However, mRNA technology requires ultracold transportation, which limits the feasibility for communities lacking cold chain facilities.[4] In contrast to mRNA vaccines, DNA molecules possess high stability and do not require ultracold storage and distribution[7]; nevertheless, the assistance of either a viral vector to deliver genetic materials to the body's cells or an electroporation device into the muscle or skin of the host is necessary to achieve efficacious DNA vaccine delivery and protective immunity against SARS-CoV-2 infection.[8–10] Such systems face the obstacle that the use of viral vectors generally elicits limited transgene capacity and broad preexisting immunity in humans. However, the electroporation device often results in substantial cell death caused by high-voltage pulses. Therefore, it should be very interesting to extend the range of LNP systems to DNA vaccine delivery and immunogenicity.

The LNP commonly contains four hybrid lipid components, and each component makes a contribution to structural stability and functional activity.[4,11] The components include cholesterol (for cell transfection), phospholipid (a helper lipid for particle structure), ionizable cationic lipid (for cellular uptake and endosomal escape of NA), and PEGylated lipid (for LNP stability/circulation). Cholesterol and phospholipids are building block components embedded in the lipid bilayer of the cell membrane.[4] The structural function of ionizable cationic lipids is the key factor in the expression kinetics

Received 19 January 2024; accepted 14 June 2024;
https://doi.org/10.1016/j.omtn.2024.102261.

[5]These authors contributed equally

Correspondence: Shih-Jen Liu, National Institute of Infectious Diseases and Vaccinology, National Health Research Institutes, Miaoli 35053, Taiwan.
E-mail: levent@nhri.edu.tw
Correspondence: Ming-Hsi Huang, National Institute of Infectious Diseases and Vaccinology, National Health Research Institutes, Miaoli 35053, Taiwan.
E-mail: huangminghsi@nhri.edu.tw

© 2024 The Authors. Published by Elsevier Inc. on behalf of The American Society of Gene and Cell Therapy. This is an open access article under the CC BY license (http://creativecommons.org/licenses/by/4.0/).

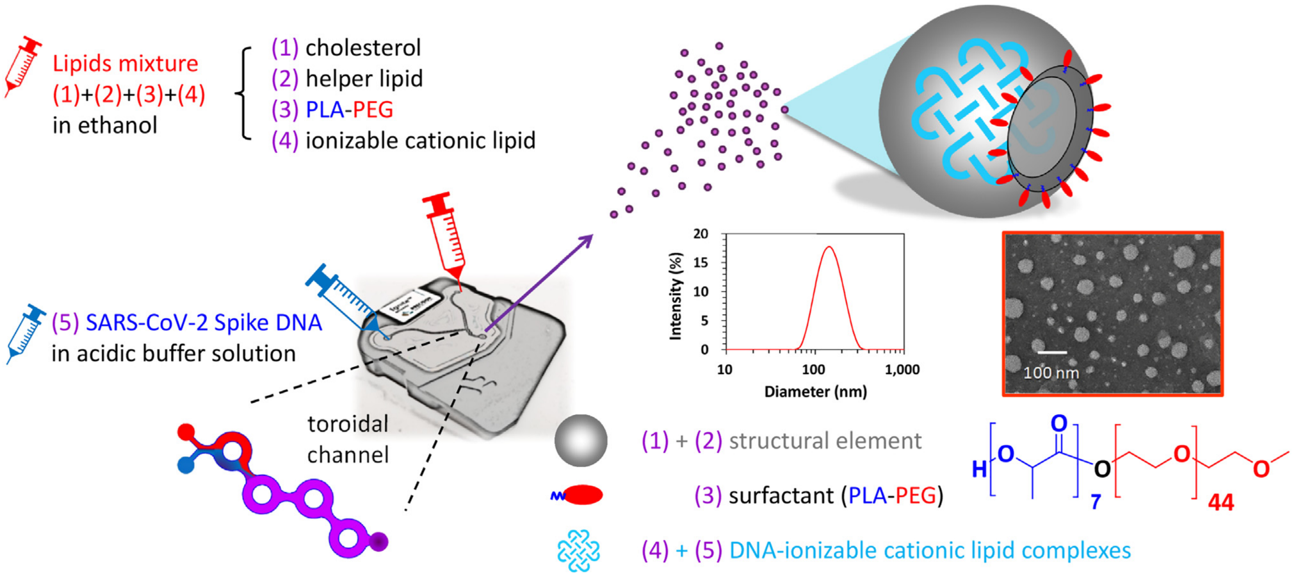

Figure 1. Self-assembled DNA-LNP vaccine

Typically, 2 miscible fluids, an aqueous solution of DNA and an organic solution comprising mixtures of lipids dissolved in ethanol, were separately pumped into the Y-shaped inlet channel of the microfluidic chip. A series of toroidal channel mixers induces chaotic advection, so the lipid components and DNA molecules can diffuse and self-assemble between ethanol-water interfaces under laminar flow conditions. Generally, cholesterol and phospholipids play a structural role in LNPs, while ionizable cationic lipid (SM-102) enables the capture of DNA in aqueous solutions and the formation of DNA-lipid complexes. In addition, the amphiphilic bioresorbable copolymer PLA-PEG serves as a surface engineering agent (surfactant) to stabilize the lipid core and aqueous phase, yielding DNA-LNPs with uniform structures at nanoscale size under DLS and TEM images.

and endosomal escape activities of NA antigen, which are associated with vaccine-induced adaptive immune responses.[11] Alternatively, PEGylated lipids act as surface engineering agents (surfactants) that stand out from the surface of LNPs.[4] These lipids reduce surface free energy between the lipophilic core and aqueous phase as well as nonspecific binding to proteins by steric repulsion. The obtained LNPs entrap and protect the designed DNA from enzymatic degradation during systemic circulation and facilitate endocytosis and endosomal escape.[4] For the first time, we repurposed an amphiphilic bioresorbable copolymer (ABC) comprising hydrophilic poly(ethylene glycol) (PEG) and lipophilic poly(lactic acid) (PLA) as an alternative to PEGylated lipids together with cholesterol, a helper lipid (phospholipid), and an ionizable cationic lipid to prepare hybrid LNPs. The immunogenicity of the DNA-LNP vaccines was determined in hamsters for the induction of protective immunity against heterologous virus challenge or as a heterologous boosting tool against SARS-CoV-2 variants.

RESULTS

Self-assembled DNA-LNP vaccine

The formation of the DNA-LNP vaccine involves a self-assembling process between two fluid streams: an acidic buffer solution of DNA encoding protein antigen and a water-miscible solution comprising the lipid mixture.[5] Automated microfluidic systems have offered a robust tool for homogenizing processes and controlling the synthesis of nanoparticles in a versatile and reproducible manner (Figure 1). This apparatus, equipped with a series of toroidal channel mixers, induces chaotic advection, so the lipid components (dissolved in ethanol) and genetic molecules (dissolved in acidic buffer solution) can diffuse and self-assemble between ethanol-water interfaces.[5,6] Typically, DNA-LNP assemblies were uniform at nanoscale as measured by dynamic light scattering (DLS) technologies. The prepared DNA-LNPs showed a spherical shape with nanostructures under transmission electron microscopy (TEM) images. Notably, the process is spontaneous—in other words, no extra energy is required to produce the nanoparticles. An efficient workflow can be achieved by steady pressure-driven flow streams such that the process was operated by controlling the total flow rate and the mixing ratio under laminar flow conditions.

Formulation efficiency and particle size are important physicochemical characteristics of DNA-LNPs, and determining these characteristics is fundamentally important for rational vaccine design. The work started by studying the effect of the content of PEGylated lipid on the prospective efficiency of LNPs (Figure 2). After passing through the inlet channel of the microfluidic chip, stable nanoparticles of uniform distribution (namely DNA-LNP-FM) can be prepared with a predetermined cholesterol:DSPC (1,2-distearoyl-*sn*-glycero-3-phosphocholine):SM-102 (8-[(2-hydroxyethyl)[6-oxo-6-(undecyloxy)hexyl]amino]-octanoic acid, 1-octylnonyl ester): DMG-PEG (1,2-dimyristoyl-rac-glycero-3-methoxy-PEG-2000) molar ratio of 38.5:10:50:1.5. By removing the DMG-PEG, a non-degradable lipid, the DNA-LNP (DNA-LNP-F1) conserved the encapsulation efficiency (EE) of LNPs, while the DNA recovery rate (RR) was

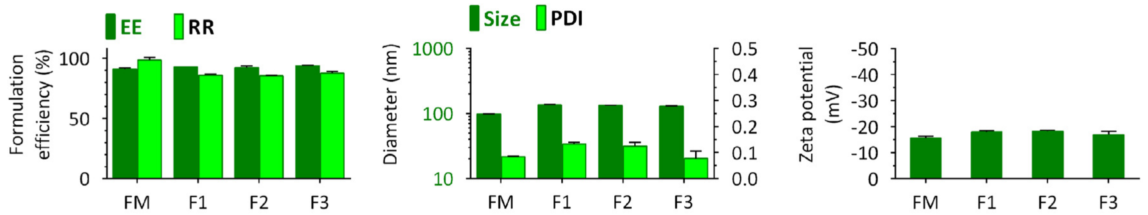

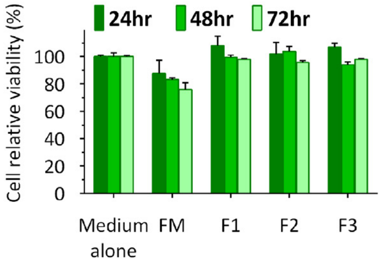

Acronym	Molar ratio of lipids			
	Cholesterol	DSPC	SM-102	PEGylated lipid
DNA-LNP-FM	38.5	10	50	1.5 [a]
DNA-LNP-F1	38.5	10	50	0
DNA-LNP-F2	38.5	10	50	1.5 [b]
DNA-LNP-F3	38.5	10	50	3.0 [b]

[a] PEGylated lipid: DMG-PEG 2000; [b] PEGylated lipid: PLA-PEG 2000

Figure 2. Impact of PEGylated lipid on the physicochemical characteristics and biocompatibility of DNA-LNPs

TSomi DNA-LNPs were characterized in terms of EE%, RR%, z-average particle size (nm), PDI, and zeta potential (mV), respectively. The biocompatibility of DNA-LNPs was conducted by the treatment of HEK293 cells with DNA-LNPs, followed by the assessment of cell viability. DNA-LNP-F1, -F2, -F3, and -FM represent DNA-LNPs without PEGylated lipid or with 1.5% PLA-PEG, 3.0% PLA-PEG, or 1.5% DMG-PEG, respectively. The cell relative viability was calculated as the percentage viability compared to medium control group at each time point. Data are represented as the mean ± SD of 3 replicates of each sample. The results are representative of 3 independent experiments.

lower (∼10%); in addition, the DNA-LNPs tended to aggregate with a broader particle size scale, polydispersity, and zeta potential. Supplementation with the appropriate ingredients of PLA-PEG helps DNA-LNPs stabilize and shrink the particles as more building block components are embedded in the LNP structure. DNA-LNP-F2 and DNA-LNP-F3 represent DNA-LNPs with 1.5% PLA-PEG and 3.0% PLA-PEG, respectively. Afterward, we investigated the biocompatibility of DNA-LNPs; this can be conducted via the treatment of HEK293 cells with DNA-LNPs, followed by assessment of cell viability. Within the 72-h incubation period, the treatment of DNA-LNP-FM induced a higher percentage of cell death in HEK293 cells compared to medium alone at each time point. Interestingly, the potency of cell death was reduced when the surface-active agents within the LNP was replaced as PLA-PEG. Collectively, we identified the cell death effects of DNA-LNPs on cells and suggested that the selection of surface-active agents may play a crucial role in DNA-LNP-induced cell death. A detailed description of the NA fundamental characterization and transfection efficiency in the cell culture study and *in vivo* distribution assessment of PLA-PEG-surface engineered DNA-LNPs is summarized in Figures S1–S5, using plasmid DNA encoding EGFP and/or green click beetle luciferase protein (CBGr99) as a payload reporter. In Figure S2, we observed that there was no or low fluorescence signal in the free DNA group, indicating low transfection. Notably, low transfection efficiency can be overcome after encapsulation into LNPs or with conventional Lipofectamine 2000 transfection reagent. However, Lipofectamine treatment induced a higher percentage of cell death in HEK293 cells compared to the groups of DNA-LNP and free DNA. Similar to the experiments with GFP-encoding DNA, quantification of mRNA transcription and protein expression was investigated in HEK293 cells during 72-h post-transfection with luciferase-encoding DNA. As shown in Figure S3A, there was no or low luciferase mRNA transcription in the free DNA group. In contrast, there was a dramatic increase in mRNA transcription after encapsulation into LNPs at each time point (24, 48, and 72 h). Interestingly, higher luciferase activity was observed for DNA-LNP formulation compared to the conventional transfection reagent. *In vivo* distribution assessment in mice with plasmid DNA encoding luciferase protein showed that the luminescence signals were durable and long lasting for at least 1 month (Figure S3B). Notably, we found that PEGylated lipid contents did not play a critical role in the transfection of DNA-LNP formulation in the cell culture system (Figure S4), as they act as surface modification agents (surfactants) rather than serving a structural function in the expression kinetics and endosomal escape activities of the DNA molecule. It is interesting to note that upon storage at 37°C, the diameter of PLA-PEG-surface engineered DNA-LNP formulations remained unchanged, and the luminescence signal produced by the cells decreased the least. However, the diameters of the DNA-LNP formulations increased upon storage at 4°C, and the luminescent signal produced by cells decreased the most (Figure S5).

Other parameters, such as the contents of the DNA, the helper lipid (DSPC), the ionizable cationic lipid (SM-102), and the ratio of PEGylated lipid to helper lipid (P/H), affect the EE and structural stability of the LNPs (Figure 3). As can be observed, in increasing the DNA content in LNPs, the DNA-LNPs tended to aggregate with a broader particle size distribution and lower DNA EE and RR. When the DNA-LNPs are prepared with different DSPC concentrations of fixed cholesterol/SM-102/PLA-PEG ratios, higher EE and

A

Acronym	DNA content (μg)	LNP composition (molar ratio) Cholestorol	DSPC	SM-102	PLA-PEG
DNA-50	50	38.5	10.0	50	1.5
DNA-66	66	38.5	10.0	50	1.5
DNA-100	100	38.5	10.0	50	1.5
DNA-200	200	38.5	10.0	50	1.5
DSPC-0	100	38.5	0.0	50	1.5
DSPC-5	100	38.5	5.0	50	1.5
DSPC-10	100	38.5	10.0	50	1.5
DSPC-20	100	38.5	20.0	50	1.5
SM-102-20	100	38.5	10.0	20	1.5
SM-102-30	100	38.5	10.0	30	1.5
SM-102-40	100	38.5	10.0	40	1.5
SM-102-50	100	38.5	10.0	50	1.5
SM-102-60	100	38.5	10.0	60	1.5
P/H-2.5/9	100	38.5	9.0	50	2.5
P/H-2/9.5	100	38.5	9.5	50	2.0
P/H-1.5/10	100	38.5	10.0	50	1.5
P/H-1/10.5	100	38.5	10.5	50	1.0
P/H-0.5/11	100	38.5	11.0	50	0.5

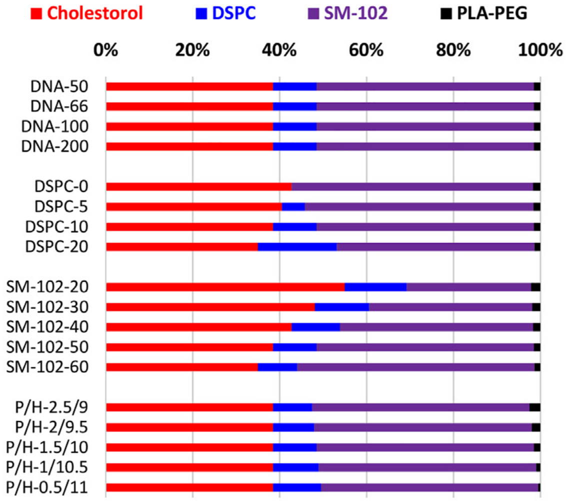

B

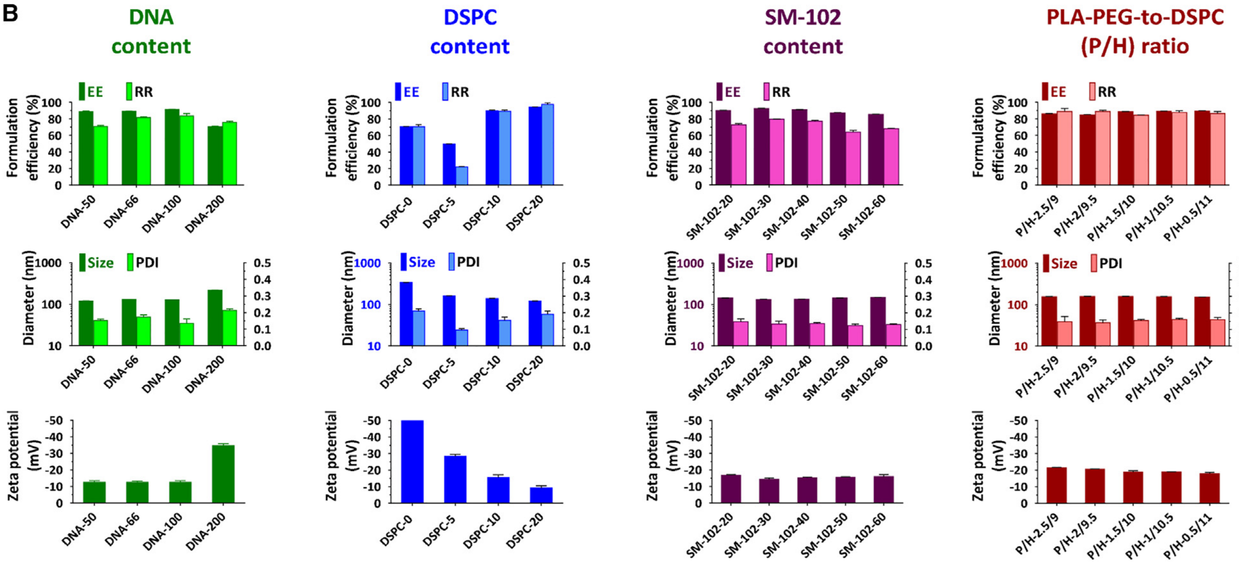

Figure 3. Parameter tuning on the physicochemical characteristics of DNA-LNPs

(A) Library of LNP chemical constituents. (B) TSomi DNA-LNPs were characterized in terms of EE%, RR%, z-average particle size (nm), PDI, and zeta potential (mV), respectively. The results are represented as the mean ± SD of 3 replicates of each sample. The standard molar ratio of lipids was cholesterol:DSPC:SM-102:PLA-PEG of 38.5:10:50:1.5, with a fixed cholesterol content of 516 μg.

RR are obtained only when the DSPC molar ratio is higher than 10. The DNA-LNPs present a smaller particle size and zeta potential when DSPC increases, as more building block components are embedded in the LNP structure. Increasing the DNA content could enlarge the particles, while increasing the DSPC content could shrink the particles. By adjusting SM-102 content could effectively drive DNA recovery and lead to property changes in LNPs. Last but not least, although PLA-PEG (PEGylated component) and DSPC (helper component) play a crucial role in the fundamental fingerprints of DNA-LNPs, we did not find the impact of the P/H ratio on the EE and structural stability of the LNPs to be within the designated ranges.

COVID-19 DNA vaccine efficacy

COVID-19 vaccines based on the initial global spread of the first SARS-CoV-2 genomic sequence, Wuhan-1, are lacking in eliciting cross-protective immunity against the emergence of variants.[1,12] Since new pandemic waves of COVID-19 are exacerbated by emerging SARS-CoV-2 variants, a feasible strategy for developing broadly protective immunity is receiving an annual vaccination or boosting with occasional updates.[1,13,14] We used plasmid DNA encoding the SARS-CoV-2 Omicron variant trimeric-spike (TSomi) gene as a model antigen to elucidate the role of DNA-LNP in vaccine immunogenicity. Previously, we investigated the use of LNP as a promising platform for new-generation mRNA/DNA vaccine

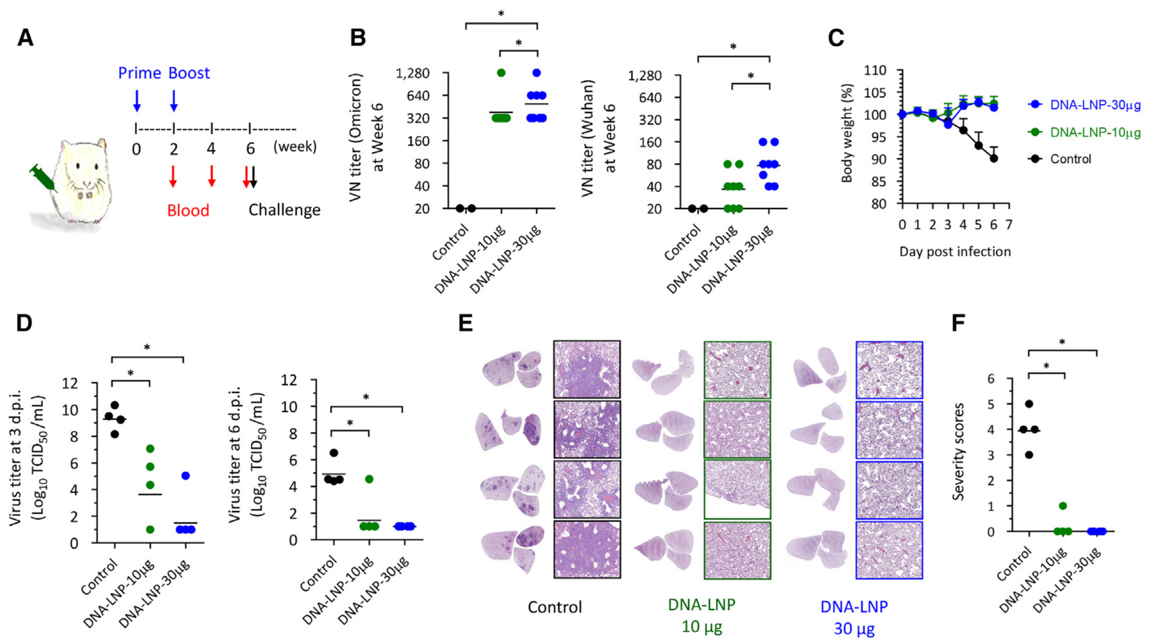

Figure 4. Vaccine efficacy of LNP-formulated DNA sequences encoding SARS-CoV-2 Omicron variant spike genes against the Wuhan original strain

(A) Schematic diagram of the vaccination regimen. Hamsters ($n = 8$) were injected with TSomi DNA-LNP through the i.m. route on day 0. At week 2, all hamsters were boosted through i.m. administration of the same vaccine formulations. (B) The VN antibodies in serum samples at week 6 are expressed as the individual values with the geometric mean titer (GMT). The Omicron TS-specific IgG titers are shown in Figure S6. In (C)–(F), the hamsters were challenged with Wuhan SARS-CoV-2 at week 6 post-vaccination. (C) Body weight change (%) after virus challenge. (D) Viral load in the lungs of infected hamsters at days 3 and 6 post-infection (d.p.i.). (E) Histopathologic examination of tissue sections (4 μm) from the lungs of infected hamsters was performed by staining with H&E and examined using a microscope (original magnifications, ×40 and ×400). (F) Pathologic severity was scored, and the p value was calculated by 1-way ANOVA with Tukey's multiple comparison test. $^*p < 0.05$.

technology.[14,15] The results demonstrated that LNP-encapsulated DNA could elicit higher humoral responses than those by a 10-times dosage of free DNA.[15] Here, we further investigated the immunological aspects of DNA-LNP surface engineered with an ABC. This investigation was conducted by studying the immunogenicity and efficacy profiles through intramuscular injection of DNA-LNP-F2 in hamsters, which is a useful animal model for evaluating COVID-19 vaccines.[3] DNA-LNP-F2 was chosen as it is a compromise between transfection efficacy and biocompatibility. It should be noted that LNP-F2 possesses the same molar ratio of lipid mixture as an approved LNP-based mRNA vaccine (LNP-FM in the present study), with the modification that the surface-active agent (DMG-PEG) has been replaced with an equivalent amount of PLA-PEG.

We conducted animal experiments on the efficiency of DNA-LNP vaccines against SARS-CoV-2 and its variants. Hamsters were administered prime-boost doses of 10 and 30 μg TSomi DNA-LNP, respectively. Tris buffer was used as the negative control. Serum samples were collected at predetermined time points for the evaluation of specific immunoglobulin G (IgG) antibody responses and protective immunity against both homologous and heterologous virus strains of SARS-CoV-2. Following the sequential vaccination regimen represented in Figure 4A, we found that the sera obtained from TSomi DNA-LNP-vaccinated hamsters could generate effective Omicron-specific neutralizing antibodies (Figure 4B, left) and IgG antibodies (Figure S6), indicating the merit of the vaccination strategy in COVID-19 protection. Interestingly, the same serum samples could neutralize Wuhan virus infection; even the neutralizing antibody titers were rather reduced (Figure 4B, right). These data are similar to the previous reports in which sera obtained from the hosts received Omicron-specific mRNA/DNA or protein vaccines possessed low cross-neutralization antibody titers against the Wuhan virus.[14–16] We subsequently investigated whether TSomi DNA-LNP could induce protective immunity in hamsters when challenged with the Wuhan strain virus. Notably, Wuhan strain virus infection induced severe disease in human and hamster animal models.[14,15] It is therefore interesting to investigate whether the TSomi DNA vaccine could induce protective immunity against Wuhan strain virus challenge. After infection with Wuhan virus, we found that the hamsters vaccinated with TSomi DNA-LNP did not show a decrease in body weight during 6 days of monitoring at either 10 or 30 μg dosages; however, the hamsters that received Tris buffer alone showed a 10% reduction in body weight (Figure 4C). As depicted in Figure 4D, the viral load in the lung showed a mild reduction on day 3 in the 10-μg DNA-LNP group compared to the buffer control, and this reduction continued to progress through day 6. However, it is important that the loaded virus was not fully eradicated from the host at this dosage. It is worth noting that increasing the dosage to 30 μg DNA-LNP effectively generated valuable viral load attenuation characteristics. The

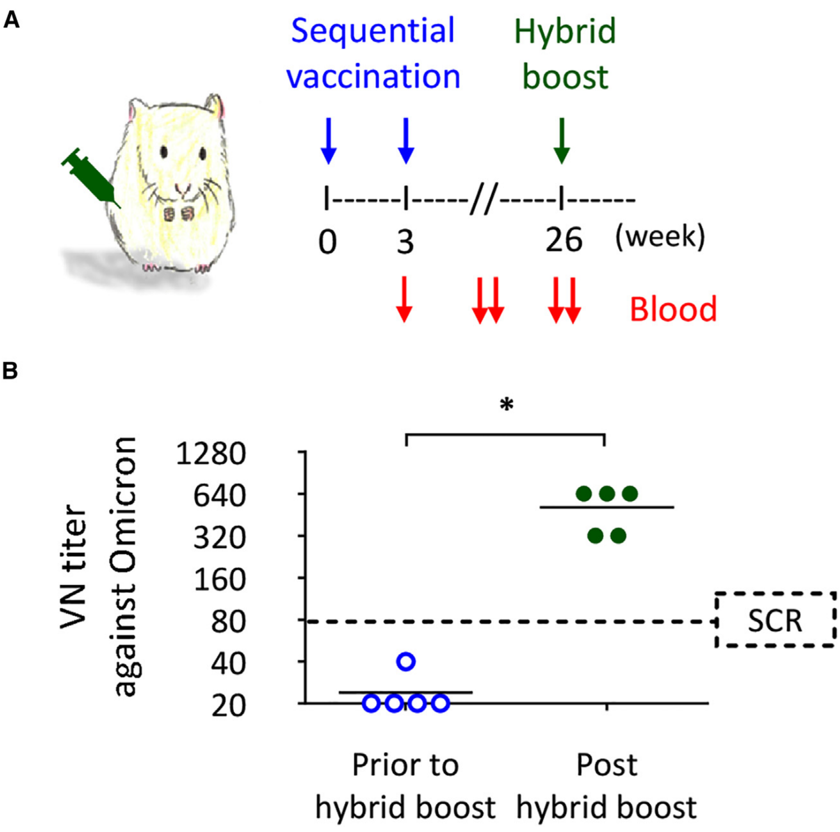

Figure 5. Boost effect of TSomi DNA-LNP on Omicron-specific VN antibodies in hamsters that were previously injected with 2 doses of Wuhan mRNA-LNP

(A) Schematic diagram of the vaccination regimen. Hamsters (n = 5) were injected with 10 μg Wuhan mRNA-LNP through the i.m. route on days 0 and 21. The Wuhan TS-specific IgG titers are shown in Figure S7. At week 26, the hamsters were boosted through i.m. administration of 10 μg TSomi DNA-LNP. (B) The Omicron-specific VN antibodies in serum samples prior to booster vaccination and 2 weeks post-booster are expressed as the individual values with the GMT. The dotted horizontal line represents seroconversion (SCR). $^{*}p < 0.05$.

pathogenesis in the lung at day 6 was analyzed, and based on the data, the buffer control group showed severe inflammation, while very low inflammation was found in the TSomi DNA-LNP vaccination groups (Figures 4E and 4F). Taken together, these data revealed that the TSomi DNA-LNP vaccine is capable of inducing protective immunity against a Wuhan virus challenge.

We next aimed to evaluate whether Omicron-specific antibodies can be driven by a hybrid-type vaccine booster. As illustrated in Figure 5A, hamsters were first administered two doses of Wuhan mRNA-LNP vaccine and generated Wuhan-specific IgG antibodies, as shown in Figure S7. At week 26, the hamsters were boosted with an Omicron-specific DNA-LNP vaccine. As shown in Figure 5B, boosting with the TSomi DNA-LNP vaccine resulted in a robust neutralizing titer against the Omicron variant, with all five hamsters exhibiting titers exceeding 80 (seroconversion). This finding implied that a boosting dose of TSomi DNA-LNP could offer protective efficacy against virus challenge.

DISCUSSION

The first generation of LNP carriers has been elaborated from proprietary lipids. Some of these components have been used as excipients in mRNA vaccines against COVID-19 and small interfering RNA (siRNA) treatment against polyneuropathy with US Food and Drug Administration (FDA) approval.[4] While allergic reactions to vaccines are rare in clinics trials, lipid components used in conventional LNP formulations are speculatively implicated in some severe cases, such as anaphylaxis.[17,18] In fact, the US Centers for Disease Control and Prevention and the US FDA recommend that individuals with a history of severe allergic reactions to any component of these vaccines/drugs refrain from receiving mRNA COVID-19 vaccines.[17] In regard to this, a growing interest in designing booster doses has spurred efforts to increase the number of well-defined lipids in the preparation of LNP-formulated genetic vaccines. Although recent studies have reported on a series of novel ionizable cationic lipids or helper lipids for screening nanoparticle-based DNA delivery,[19,20] there is a lack of in-depth analysis on the impact of the choice of PEGylated lipids and the relative proportions of lipid ingredients on the transfection efficiency and safety aspects of DNA vaccines. The present work is the first investigation on surface engineering of DNA-LNPs using diblock PLA-PEG copolymer as an alternative to PEGylated lipids. In this form, high levels of gene regulation and cellular transfection of DNA-LNPs were found in HEK293 cells; in contrast, substantial protein expression was found for free DNA. Dual-function PLA-PEG makes the obtained nanostructured lipid carrier an LNP of great versatility. Because of stability, PLA-PEG is known to be an AB-type ABC used for the design of sustained drug/vaccine delivery systems that have received great attention because they offer structure-function relationships in response to temperature stimuli.[21] This system could help further design an optimal vesicle for NA delivery that does not require cold chain storage and distribution. Regarding biocompatibility, we suggest using PEG segments of molecular weight 2,000 on the one hand, as it possesses beneficial properties, including the steric hindrance and clearance of LNPs[4]; on the other hand, PLA was chosen as the lipophilic block, as it provides a worthwhile way to ensure the absorbability of DNA-LNPs during post-vaccination.[21] As a result, designer vaccine LNPs obtained worthwhile bioresorbable nanoparticles that help reduce the risk of side effects and adverse events.

In the present study, the results from virus infection showed that Omicron-specific DNA-LNP vaccine candidates can induce protective immunity against Wuhan strain SARS-CoV-2 challenge (Figure 4). We previously used angiotensin-converting enzyme 2 transgenic mice to demonstrate that such cross-protective immunity is mediated by $CD8^+$ T cells.[14] Interestingly, we also demonstrated that immunization of DNA provided greater protection than mRNA immunization in hamsters, using the same dosage and LNP formulation.[15] It is known that the ability to remodel the tumor microenvironment and the generation of antigen-specific $CD8^+$ T cells are crucial in successful tumor immunotherapy.[22] With this potential in mind, the anticipated research tasks could be extended for further evaluation of novel molecular therapeutic strategies into human trials, aiming to generate therapeutic immunity against cancer. Insofar as the vaccination route was concerned, the currently approved/authorized NA-based COVID-19 vaccines are administered intramuscularly (i.m.), which fail to provide protection against SARS-CoV-2 virus replication in the nasopharynx. However, the clinical trial results showed that intranasal vaccination with viral vector DNA did not produce a consistent mucosal antibody response nor a strong systemic response.[23] To effectively deliver NA genomes to mucosa-associated lymphoid tissue, it is necessary to incorporate an immunostimulatory adjuvant. This facilitates the mucosal immune system to recognize antigens, leading to the generation of protective mucosal immune responses. Further studies are warranted to find an optimal dosage of combination adjuvant to develop a nasal spray vaccine for both mucosal activity and neutralizing antibody response.

In summary, successful DNA vaccination can be achieved with the aid of an LNP approach that comprises a hybrid mixture of cholesterol, helper lipid, ionizable cationic lipid, and an AB-type amphiphilic bioresorbable copolymer. The rational design of DNA-LNP conciliates between efficacy and safety, which offers new insights into COVID-19 vaccine boosters. The outcomes of this study will provide critical mechanistic insights into boosting the vaccine power of lipid nanoparticles and direction for the optimization of genetic vaccine formulations against possible future emerging infectious diseases/pathogens.

MATERIALS AND METHODS

Plasmid construction and characterization

A plasmid DNA encoding SARS-CoV-2 TSomi was used as a vaccine antigen in immunogenicity studies,[10,14,15] which was optimized for human codon usage and synthesized by GenScript Biotech (Piscataway, NJ), as previously described.[10] In the cell culture study and *in vivo* distribution assessment, plasmid DNA sequences encoding EGFP or CBGr99 were used as reporter genes for monitoring the transfection/expression of the DNA-LNP formula (see Figure S1 for plasmid map of pVAX1-TSomi, pVAX1-CBGr99, and pcDNA3.1-GFP, respectively). The TSomi and CBGr99 genes were subcloned into the clinically used vector pVAX1 with the Kozak sequence incorporated at the 5′ end of the genes, as previously described.[10] GFP was expressed by a pcDNA3.1 backbone plasmid vector. All plasmids were transformed into *Escherichia coli* DH5α cells (ECOS101, Yeastern Biotech, Taipei City, Taiwan) for plasmid amplification, followed by extraction and purification using an endotoxin-free Qiagen column system (EndoFree Plasmid Mega Kit, catalog no. 12381, Qiagen, Hilden, Germany).

DNA-LNP formation

Cholesterol was purchased from Sigma-Aldrich (catalog no. C3045, St. Louis, MO). SM-102 and DMG-PEG were purchased from Cayman Chemical (catalog nos. 33474 and 33945, Ann Arbor, MI). DSPC was purchased from Avanti Polar Lipids (catalog no. 850365P, Birmingham, AL). The AB-type diblock copolymer PLA-PEG with molecular characteristics of 20 wt % PLA and 80 wt % PEG was synthesized by ring-opening polymerization of lactide on PEG monomethyl ether (number average molecular weight ~2,000), as described previously.[24] DNA-LNP formation was elaborated using a microfluidic mixer system (NanoAssemblr Ignite; Precision Nanosystems, Vancouver, BC, Canada). Lipid mixtures (cholesterol:helper lipid: ionizable cationic lipid:PEGylated lipid) were dissolved in ethanol at predetermined molar ratios. DNA was dissolved in 6.25 mM sodium acetate (catalog no. 567422, EMD Millipore, Burlington MA) aqueous solution, providing an acidic environment at pH 5.2. Typically, a volumetric flow ratio was set at 1/3 (ethanol/aqueous) and a total flow rate of 12 mL/min for the mixing of two streams flowing through microfluidic channels, where a 2-mg lipid mixture (700 μL) with cholesterol/DSPC/SM-102/PLA-PEG molar composition of 38.5/10/50/1.5 was combined with 100 μg DNA-containing buffer solution (2,100 μL). The crude formulations were diluted with a total 120 mL of 20 mM Tris-HCl buffer (pH 7.5) (catalog no. 15567-027 Invitrogen; Grand Island, NY) and concentrated with Amicon Ultra centrifugal filters (cutoff: 10,000, catalog no. UFC901096 Merck, Dublin, Ireland). The recovered products were further sterilized by filtration using a 0.45-μm filter (Acrodisc syringe filters with Supor membrane, catalog no. 4604, Pall Corporation, Fajardo, Puerto Rico) and stored in Tris buffer until use. mRNA-LNP formulation was synthesized in the same manner with an equivalent amount of mRNA.

Measurements

Various analytical techniques were conducted to provide a range of fundamental information of LNP, including its surface morphology using TEM techniques (H-7650, Hitachi, Tokyo, Japan), particle size and electrophoretic mobility using light-scattering techniques (Malvern Zetasizer Pro-blue, Malvern Panalytical, Malvern, UK), and DNA determination using a fluorescence detection method (catalog no. R11490, Quant-iT RiboGreen RNA Kit, Thermo Fisher Scientific, Eugene, OR). The particle size and electrophoretic mobility were measured by DLS and electrophoretic light scattering, respectively; and data were converted to z-average particle size (diameter, nm), polydispersity index (PDI), and zeta potential (mV). EE% of the DNA in the LNP was calculated as the following equation: $EE\% = (D_0 - D_1)/D_0 \times 100$, where D_0 and D_1 represent total DNA in solution after and before LNP lysis, respectively. The RR% of DNA was calculated as the percentage of D_1 divided by the initial amount of DNA added.

In vitro transfection

A group of 24-well plates were seeded with 1×10^5 HEK293 cells (catalog no. BCRC60019) in DMEM (catalog no. SH30243.02, HyClone, Logan, UT) supplemented with 10% fetal bovine serum (catalog no. 10437-028, Gibco, Grand Island, NY) at a total volume of 1 mL per well. At 24-h post-seeding, cells were transfected in triplicate in the presence of 1 μg DNA-LNP. Cells transfected with the commercial transfection reagent TurboFect (catalog no. R0531, Thermo Fisher Scientific) or Lipofectamine 2000 (catalog no. 100014469, Thermo-Fisher Scientific) were used as a positive control. The plates were then incubated for 3 days at 37°C and 5% CO_2 in air. The cells were stained with trypan blue solution (0.4%) (catalog no. T10282, Invitrogen, Carlsbad, CA) following the supplier's instructions and analyzed with the Countess 3 FL Automated Cell Counter (Thermo Fisher Scientific). The cell viability was denoted as the cells that have clear cytoplasm. The following reporter genes were selected as indicators for transfection efficiency: EGFP and CBGr99.

Hamster vaccination and challenge

Syrian hamsters were obtained from the National Laboratory Animal Breeding and Research Center (Taipei, Taiwan), housed at the Animal Center of the National Health Research Institutes (NHRI), and maintained in accordance with institutional animal care protocols (protocol no. NHRI-IACUC-109077-A).

The vaccine immunogenicity study was conducted in 6- to 12-week-old hamsters. Two sets of experiments were performed: (1) sequential vaccination with TSomi DNA-LNP and (2) sequential vaccination with Wuhan-specific mRNA-LNP, followed by TSomi DNA-LNP boosting dose.

For the sequential DNA vaccination experiment, hamsters (n = 8) were vaccinated i.m. with two doses of vaccine candidates at a 2-week interval. Three vaccination groups were compared as follows: group 1 received Tris buffer only; group 2 received 10 μg DNA-LNP; and group 3 received 30 μg DNA-LNP. At weeks 2, 4, and 6 following vaccination, serum samples were obtained through retroorbital blood sampling to assess the Omicron-specific antibody titers. The presence of Omicron-specific IgG antibodies in the sera was determined by ELISA, as described previously.[14] The virus-neutralizing (VN) antibodies were determined as described below. Four weeks after the final vaccination, the hamsters under isoflurane anesthesia were challenged intranasally with 10^5 $TCID_{50}$ (50% tissue culture infectious dose) SARS-CoV-2 (hCoV-19/Taiwan/4/2020) in a 50-μL volume. Their body weights were monitored every day after the challenge for 6 days. Subsequently, four hamsters from each group were sacrificed on days 3 and 6 after challenge for viral load quantification and pathologic examination. The viral load was determined by homogenizing left lung tissues in 2 mL PBS using a gentleMACS Dissociator (Miltenyi Biotec, Auburn, CA). After centrifugating the lung homogenate at 600 × g for 5 min, the virus-containing supernatant was collected for live virus titration by $TCID_{50}$ assay, as previously described.[15,19]

For the hybrid-type vaccine booster experiment, hamsters (n = 5) were vaccinated i.m. with two doses of 10 μg mRNA-LNP at a 3-week interval. The production process of the mRNA encoding SARS-CoV-2 Wuhan trimeric spike was performed as previously described.[15] Briefly, mRNA was synthesized by *in vitro* transcription reaction by T7 RNA polymerase-mediated transcription from a linearized pT7TS plasmid DNA template. The final mRNA utilizes CleanCap Reagent AG 3′ OMe as 5′ cap (catalog no. *N*-7413, TriLink, San Diego, CA) and full replacement of uridine with N1-methylpseudouridine-5′-triphosphate. At week 26, the hamsters were boosted i.m. with 10 μg TSomi DNA-LNP. Serum samples were collected at predetermined time points for the evaluation of systemic antibody responses.

VN assay

For the VN assay, 200 $TCID_{50}$ per well of SARS-CoV-2 virus (Wuhan: hCoV-19/Taiwan/4/2020, Omicron: hCoV-19/Taiwan/16804/2021) were incubated with 2-fold-diluted hamster sera at a starting dilution of 1:40 with M199 medium (Gibco, catalog no. 11150059). Mixtures of virus and serum were transferred to monolayers of Vero cells and incubated at 37°C and 5% CO_2 for 4 days. The neutralizing titer was defined as the reciprocal of the highest serum dilution at which the infectivity of the SARS-CoV-2 virus at 200 $TCID_{50}$ for Vero cells was completely neutralized in 50% of quadruplicate inoculations. Infectivity was identified by the presence of cytopathy after 4–5 days of incubation, and the neutralization titer was calculated using the Reed-Muench method. For calculation purposes, an undetectable level was scored as a titer equal to 20.

H&E staining and pathologic scoring

The lung tissue samples of infected hamsters were excised, fixed with 4% paraformaldehyde overnight, and embedded in paraffin by a routine histopathological examination method performed by the core pathology facility at the NHRI. We stained 4-μm sections with H&E and analyzed using a Leica DFC 5400 digital camera. The captured images were processed using Leica Application Suite version 4.13. To quantify the severity of lung tissue damage, the pathologic score was scored as described in a previous study.[14]

Statistical analysis

The statistically significant difference between each vaccination group was assessed by GraphPad Prism software. The viral load in the lungs of infected hamsters and the pathologic severity scoring of lung sections after viral challenge were determined, and the p value was calculated by an ANOVA model with Tukey's multiple comparison test. The antibody titers were compared by performing log-transformed values. The comparison of antibodies before and after the boost was performed by a two-tailed paired t test. A value of 0.05 was considered an indication of significance.

DATA AND CODE AVAILABILITY

The findings of this study are available within this paper or are available from the corresponding author upon reasonable request.

SUPPLEMENTAL INFORMATION

Supplemental information can be found online at https://doi.org/10.1016/j.omtn.2024.102261.

ACKNOWLEDGMENTS

This work was supported by the NHRI of Taiwan (IV-113-PP-18 and IV-112-SP-A7) and by grants from the National Science and Technology Council of Taiwan (NSTC 112-2314-B-400-023 and NSTC 112-2740-B-400-004). The authors are grateful to NSTC for awarding a postdoctoral research scholarship to C.-H.Y. (NSTC 112-2811-B-400-026). The authors are grateful to the team members of the P3 facility, Bioproduction Plant of NHRI, for conducting the VN assays and challenge tests. The authors gratefully acknowledge Miss Laetitia Le-Ping Huang for her help with graphic artwork.

AUTHOR CONTRIBUTIONS

Conceptualization, H.-W.C., C.-L.L., S.-J.L., and M.-H.H. Methodology: C.-H.Y., K.-Y.S., H.-M.H., C.-Y.H., Y.-J.C., C.-C.P., F.-F.C., W.-C.H., and H.-C.L. Investigation: C.-H.Y., K.-Y.S., H.-M.H., and C.-Y.H. Visualization: C.-H.Y., K.-Y.S., H.-M.H., and C.-Y.H. Funding acquisition: S.-J.L. and M.-H.H. Project administration: S.-J.L. and M.-H.H. Supervision: H.-W.C., C.-L.L., S.-J.L., and M.-H.H. Writing – original draft: C.-H.Y. and K.-Y.S. Writing – review & editing: S.-J.L. and M.-H.H.

DECLARATION OF INTERESTS

The authors declare no competing interests.

REFERENCES

1. Subbarao, K. (2021). The success of SARS-CoV-2 vaccines and challenges ahead. Cell Host Microbe *29*, 1111–1123.
2. Callaway, E. (2020). The race for coronavirus vaccines: a graphical guide. Nature *580*, 576–577.
3. Lurie, N., Saville, M., Hatchett, R., and Halton, J. (2020). Developing Covid-19 vaccines at pandemic speed. N. Engl. J. Med. *382*, 1969–1973.
4. Hou, X., Zaks, T., Langer, R., and Dong, Y. (2021). Lipid nanoparticles for mRNA delivery. Nat. Rev. Mater. *6*, 1078–1094.
5. Molinaro, R., Evangelopoulos, M., Hoffman, J.R., Corbo, C., Taraballi, F., Martinez, J.O., Hartman, K.A., Cosco, D., Costa, G., Romeo, I., et al. (2018). Design and development of biomimetic nanovesicles using a microfluidic approach. Adv. Mater. *30*, e1702749.
6. Stroock, A.D., Dertinger, S.K.W., Ajdari, A., Mezic, I., Stone, H.A., and Whitesides, G.M. (2002). Chaotic mixer for microchannels. Science *295*, 647–651.
7. Algarni, A., Pilkington, E.H., Suys, E.J.A., Al-Wassiti, H., Pouton, C.W., and Truong, N.P. (2022). In vivo delivery of plasmid DNA by lipid nanoparticles: the influence of ionizable cationic lipids on organ-selective gene expression. Biomater. Sci. *10*, 2940–2952.
8. Tebas, P., Yang, S., Boyer, J.D., Reuschel, E.L., Patel, A., Christensen-Quick, A., Andrade, V.M., Morrow, M.P., Kraynyak, K., Agnes, J., et al. (2021). Safety and immunogenicity of INO-4800 DNA vaccine against SARS-CoV-2: A preliminary report of an open-label, Phase 1 clinical trial. EClinicalMedicine *31*, 100689.
9. Chai, K.M., Tzeng, T.T., Shen, K.Y., Liao, H.C., Lin, J.J., Chen, M.Y., Yu, G.Y., Dou, H.Y., Liao, C.L., Chen, H.W., et al. (2023). DNA vaccination induced protective immunity against SARS CoV-2 infection in hamsters. PLoS Neglected Trop. Dis. *17*, e0011045.
10. Tzeng, T.T., Chai, K.M., Shen, K.Y., Yu, C.Y., Yang, S.J., Huang, W.C., Liao, H.C., Chiu, F.F., Dou, H.Y., Liao, C.L., et al. (2022). A DNA vaccine candidate delivered by an electroacupuncture machine provides protective immunity against SARS-CoV-2 infection. NPJ Vaccines *7*, 60.
11. Chaudhary, N., Weissman, D., and Whitehead, K.A. (2021). mRNA vaccines for infectious diseases: principles, delivery and clinical translation. Nat. Rev. Drug Discov. *20*, 817–838.
12. Afkhami, S., D'Agostino, M.R., Zhang, A., Stacey, H.D., Marzok, A., Kang, A., Singh, R., Bavananthasivam, J., Ye, G., Luo, X., et al. (2022). Respiratory mucosal delivery of next-generation COVID-19 vaccine provides robust protection against both ancestral and variant strains of SARS-CoV-2. Cell *185*, 896–915.e19.
13. Fang, Z., Peng, L., Filler, R., Suzuki, K., McNamara, A., Lin, Q., Renauer, P.A., Yang, L., Menasche, B., Sanchez, A., et al. (2022). Omicron-specific mRNA vaccination alone and as a heterologous booster against SARS-CoV-2. Nat. Commun. *13*, 3250.
14. Shen, K.Y., Yang, C.H., Chen, C.T., Ho, H.M., Chiu, F.F., Huang, C.Y., Liao, H.C., Hsu, C.W., Yu, G.Y., Liao, C.L., et al. (2023). Omicron-specific mRNA vaccine induced cross-protective immunity against ancestral SARS-CoV-2 infection with low neutralizing antibodies. J. Med. Virol. *95*, e28370.
15. Liao, H.C., Shen, K.Y., Yang, C.H., Chiu, F.F., Chiang, C.Y., Chai, K.M., Huang, W.C., Ho, H.M., Chen, Y.H., Huang, M.S., et al. (2024). Lipid nanoparticle-encapsulated DNA vaccine robustly induce superior immune responses to the mRNA vaccine in Syrian hamsters. Mol. Ther. Methods Clin. Dev. *32*, 101169.
16. Kingstad-Bakke, B., Lee, W., Chandrasekar, S.S., Gasper, D.J., Salas-Quinchucua, C., Cleven, T., Sullivan, J.A., Talaat, A., Osorio, J.E., and Suresh, M. (2022). Vaccine-induced systemic and mucosal T cell immunity to SARS-CoV-2 viral variants. Proc. Natl. Acad. Sci. USA *119*, e2118312119.
17. Banerji, A., Wickner, P.G., Saff, R., Stone, C.A., Blumenthal, K.G., Jr., Robinson, L.B., Long, A.A., Wolfson, A.R., Williams, P., Khan, D.A., and Phillips, E. (2021). mRNA vaccines to prevent COVID-19 disease and reported allergic reactions: Current evidence and suggested approach. J. Allergy Clin. Immunol. Pract. *9*, 1423–1437.
18. Sprent, J., and King, C. (2021). COVID-19 vaccine side effects: The positives about feeling bad. Sci. Immunol. *6*, eabj9256.
19. Guimaraes, L.C., Costa, P.A.C., Scalzo Júnior, S.R.A., Ferreira, H.A.S., Braga, A.C.S., de Oliveira, L.C., Figueiredo, M.M., Shepherd, S., Hamilton, A., Queiroz-Junior, C.M., et al. (2024). Nanoparticle-based DNA vaccine protects against SARS-CoV-2 variants in female preclinical models. Nat. Commun. *15*, 590.
20. Zhu, Y., Shen, R., Vuong, I., Reynolds, R.A., Shears, M.J., Yao, Z.C., Hu, Y., Cho, W.J., Kong, J., Reddy, S.K., et al. (2022). Multi-step screening of DNA/lipid nanoparticles and co-delivery with siRNA to enhance and prolong gene expression. Nat. Commun. *13*, 4282.
21. Jeong, B., Bae, Y.H., Lee, D.S., and Kim, S.W. (1997). Biodegradable block copolymers as injectable drug-delivery systems. Nature *388*, 860–862.
22. Baharom, F., Ramirez-Valdez, R.A., Khalilnezhad, A., Khalilnezhad, S., Dillon, M., Hermans, D., Fussell, S., Tobin, K.K.S., Dutertre, C.A., Lynn, G.M., et al. (2022). Systemic vaccination induces $CD8^+$ T cells and remodels the tumor microenvironment. Cell *185*, 4317–4332.e15.
23. Madhavan, M., Ritchie, A.J., Aboagye, J., Jenkin, D., Provstgaad-Morys, S., Tarbet, I., Woods, D., Davies, S., Baker, M., Platt, A., et al. (2022). Tolerability and immunogenicity of an intranasally-administered adenovirus-vectored COVID-19 vaccine: An open-label partially-randomised ascending dose phase I trial. EBioMedicine *85*, 104298.
24. Chen, W.L., Liu, S.J., Leng, C.H., Chen, H.W., Chong, P., and Huang, M.H. (2014). Disintegration and cancer immunotherapy efficacy of a squalane-in-water delivery system emulsified by bioresorbable poly(ethylene glycol)-block-polylactide. Biomaterials *35*, 1686–1695.

OMTN, Volume *35*

Supplemental information

Boosting DNA vaccine power by lipid nanoparticles surface engineered with amphiphilic bioresorbable copolymer

Chung-Hsiang Yang, Kuan-Yin Shen, Hui-Min Ho, Chiung-Yi Huang, Yu-Jhen Cheng, Chih-Chun Pu, Fang-Feng Chiu, Wan-Chun Huang, Hung-Chun Liao, Hsin-Wei Chen, Ching-Len Liao, Shih-Jen Liu, and Ming-Hsi Huang

Supplemental methods

***In vitro* imaging.** After 72-hr transfection with GFP DNA-LNPs, the transfected cells were cultured in 24-well plates (Cat. #142475, Nunc, Thermo-Fisher Scientific, China) in 1 mL culture medium, and images were taken with a fluorescence microscope (Olympus IX73 inverted microscope, Tokyo, Japan). Cell nuclei were stained with Hoechst 33342 dye (Thermo-Fisher Scientific, Rockford, IL, USA) before imaging.

***In vitro* luminescence.** After 72-hr transfection with CBGr99 DNA-LNPs, the transfected cells were harvested and washed with PBS and then lysed using 150 μL of Passive Lysis Buffer (Cat. #E1941, Promega, WI, USA). The luciferase activity was measured by mixing 50 μL of the cell lysate with 50 μL of luciferase substrate (Cat. #E151A, Promega, WI, USA) in a 96-well white plate (Cat. #236108, Nunc, Thermo-Fisher Scientific, Denmark) and then measuring the luminescence by an Orion L Microplate Luminometer (Berthold Detection System, TN, USA). Luciferase activities were expressed as relative light units per second (RLU/s).

RNA extraction and RT-qPCR. RNA was extracted at 24, 48, 72 hrs post-transfection using the RNeasy® Mini Kit (Cat. #74104, QIAGEN, Hilden, Germany) following the manufacturer's protocol. Prior to RNA extraction, the cell culture medium was removed, and the cells were washed once with PBS before being lysed. From the lysates (600 ng of RNA), cDNA was synthesized using SuperScript III Reverse Transcriptase (Cat. #18080044, Thermo-Fisher Scientific, MA, USA) with oligo(dT)12-18 primer (Cat. #18418012, Thermo-Fisher Scientific, MA, USA) following the manufacturer's instructions. Before qPCR reaction, cDNA samples were diluted 3 times in RNase- and DNase-free water. 1 μL cDNA was used in each reaction with 200 nM forwards and reverse primers (Integrated DNA Technologies, Singapore). PCR was performed

with KAPA SYBR® FAST qPCR Master Mix (Cat. #kk4609, KAPA BIOSYSTEMS, MA, USA) on a LightCycler® 480 (Roche Diagnostics, IN, USA) at a total volume of 10 μL in 384-well plates (Roche Diagnostics, IN, USA). The cycling conditions were set as pre-incubation at 95°C for 3 min, followed by 40 cycles of 95°C for 10 sec, 60°C for 20 sec, and 72°C for 1 sec. The program ended with melting curve analysis to check primer dimers. In the experiment, two replicates of cDNA samples were performed along with no transcriptase control or no template control to check genomic DNA contamination. Expression data were analyzed using the Delta-Delta Ct method. The expression of GAPDH was used as a reference control.

DNA-LNP stability. Stability monitoring of the DNA-LNP formulations was performed at 4, 15, 25, and 37°C. At week 2 and week 4, three specimens were withdrawn and tested for their particle size distribution, encapsulation efficiency and transfection of DNA reporter genes in HEK293 cells (human embryonic kidney 293 cells, Cat. #BCRC60019).

***In vivo* luminescence in mice.** BALB/c mice were obtained from the NLABRC (Taipei, Taiwan) and housed at the Animal Center of the NHRI and maintained in accordance with institutional animal care protocols (Protocol No: NHRI-IACUC-109077-A). Transfection of DNA molecules and expression of luciferase protein in mice were measured by using *in vivo* luminescence. Mice were inoculated with predetermined amounts of the DNA-LNP (10 μg per mouse) via the intramuscular (i.m.) route. On Days 1, 2, 3, 4, 7, 10, 14, 21, and 28 after administration, mice were injected intraperitoneally (i.p.) with 100 μL D-luciferin (Cat. # ab143655, Abcam, Cambridge, UK) at a concentration of 15 mg/mL in PBS, and the reaction were performed for 11 minutes. Luminescence signals were collected by an IVIS Spectrum instrument (Perkin Elmer, Waltham, MA, USA), and the luminescence signals in regions of interest (ROIs) were quantified using the imaging software "Living Image".

Supplemental Figures

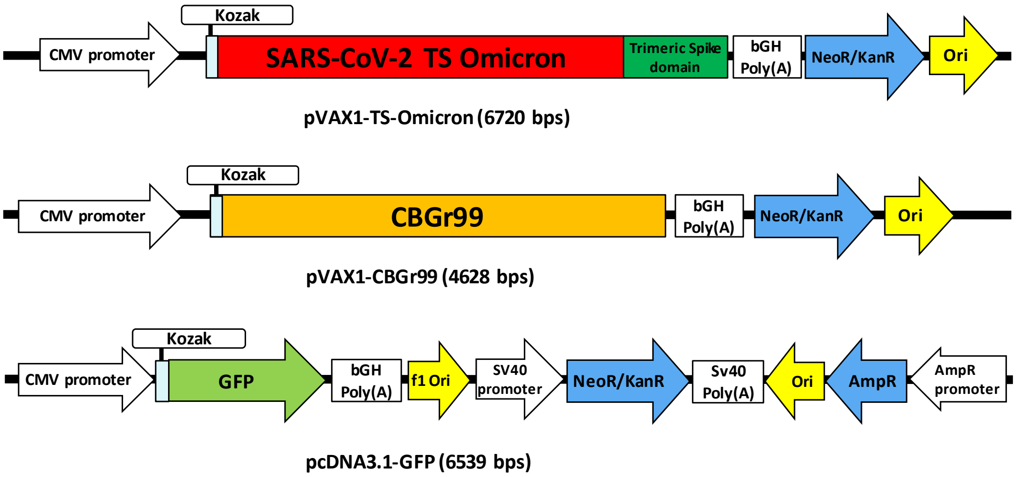

Acronym	Encapsulation efficiency (EE%)	Particle size and electrophoretic mobility		
		Diameter (Z-avg, nm)	Polydispersity index (PDI)	Zeta potential (mV)
TS DNA-LNP	91.4 ± 0.3	127.8 ± 0.4	0.135 ± 0.027	-12.5 ± 1.0
CBGr99 DNA-LNP	93.1 ± 0.2	118.4 ± 0.4	0.118 ± 0.022	-11.2 ± 1.3
GFP DNA-LNP	82.5 ± 0.4	139.2 ± 1.4	0.126 ± 0.011	-24.5 ± 0.4

Figure S1. Plasmid illustration of plasmids used in the present study and fundamental characterization of DNA-LNPs

DNA-LNPs were characterized in terms of encapsulation efficiency (EE%), Z-avg particle size (nm), polydispersity index (PDI), and zeta potential (mV). The results are represented as the mean ± STD of three samples. The molar ratio of lipids was cholesterol:DSPC:SM-102:PLA-PEG of 38.5:10:50:1.5.

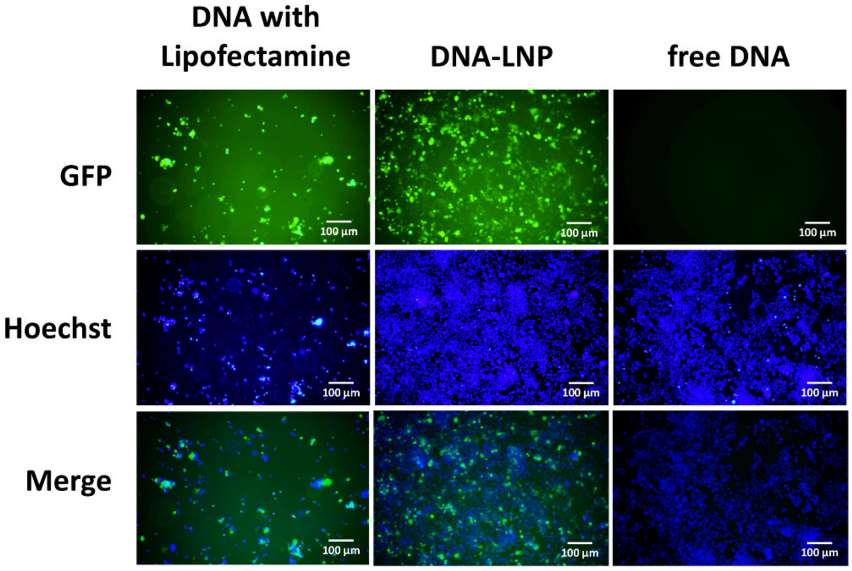

Figure S2. Effects of LNP on DNA transfection in the cell culture system

1 × 10^5 HEK293 cells were treated with GFP-encoding plasmid DNA, either in culture medium (DNA alone), encapsulated within the LNP formulation or in the presence of the conventional transfection reagent Lipofectamine™ 2000. Complexes were prepared by pre-incubating 1 µg DNA and 3 µL Lipofectamine™ 2000 in 100 µL serum-free DMEM at room temperature for 20 min following manufacturer instructions. Observation of protein expression by fluorescence microscopy (green channel) and Hoechst staining (blue channel) of cell nuclei after 72 hrs. Scale bar: 100 µm. The molar ratio of lipids was cholesterol:DSPC:SM-102:PLA-PEG of 38.5:10:50:1.5.

A

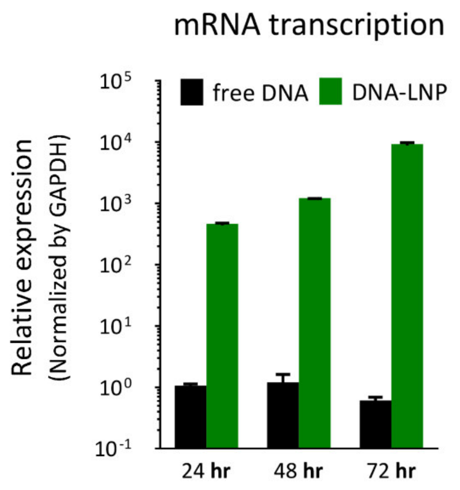

B

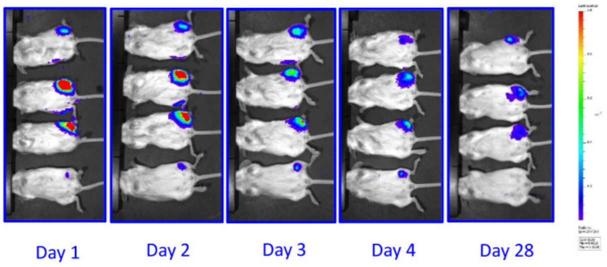

C

Gene	Primer sequence	Length
CBGr99	5'-GTGTCACCGTCTTGGTCTAC-3'<br>5'-CGCGGAGACCGACCATAAAG-3'	64 bps
GAPDH	5'-GAGAAGGCTGGGGCTCATTT-3'<br>5'-TGATGACCCTTTTGGCTCCC-3'	48 bps

Figure S3. Transfection of DNA-LNP formulation *in vitro* and *in vivo*

(A) Transcription and protein expression of the DNA-LNP formulation in the cell culture system. 1×10^5 HEK293 cells were treated with the CBGr99 luciferase plasmid, either in culture medium (free DNA) or in the LNP formulation. The molar ratio of lipids was cholesterol:DSPC:SM-102:PLA-PEG of 38.5:10:50:1.5 (CBGr99 DNA-LNP-F2). Luciferase mRNA quantification in HEK293 cells at 24-, 48-, and 72-hr post transfection was analyzed by the Delta-Delta Ct method. Luciferase activity at 72-hr post transfection was measured as relative light units (RLU/s) in protein lysates from HEK293 cells transfected with plasmid encoding CBGr99 luciferase. (B) Bioluminescence images at the site of injection in a mouse model. Four BALB/c mice were injected intramuscularly with DNA-LNPs (10 µg CBGr99 luciferase plasmid per mouse). Representative IVIS images of DNA-LNPs at selected time points. (C) Primers used for RT-qPCR analysis.

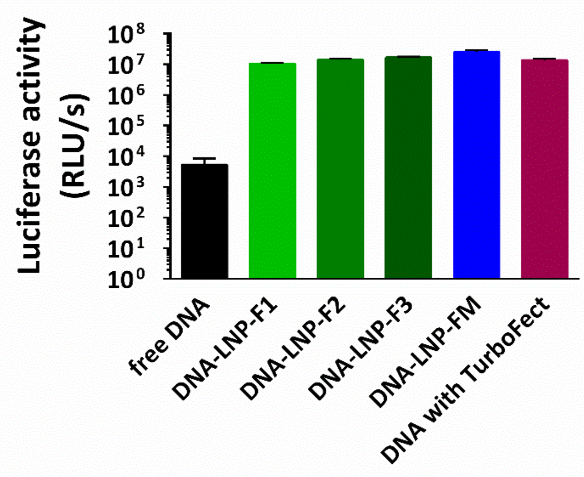

Figure S4. Impact of the lipid contents on the transfection of DNA-LNP formulation in the cell culture system

1 × 10^5 HEK293 cells were treated with the CBGr99 luciferase plasmid, either in culture medium (DNA alone), within different LNP formulation or in the presence of the conventional transfection reagent Turbofect™. Luciferase activity at 72-hr post transfection was measured as relative light units (RLU/s) in protein lysates from HEK293 cells transfected with plasmid encoding CBGr99 luciferase. DNA-LNP-F1, -F2, -F3, -FM represent DNA-LNPs without PEGylated lipid or with 1.5% PLA-PEG or 3.0% PLA-PEG, or 1.5% DMG-PEG, respectively. Data are represented as the mean ± STD of three replicates of each sample.

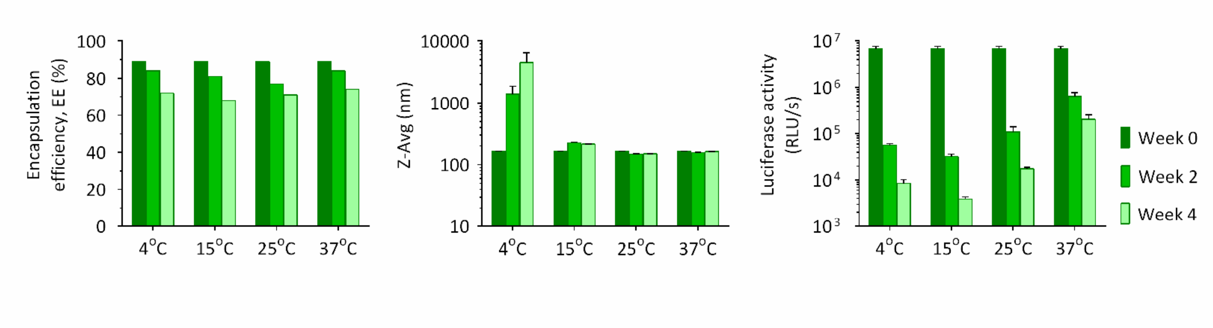

Figure S5. Characteristic changes of DNA-LNP upon storage at 4°C, 15°C, 25°C and 37°C

DNA-LNP was prepared with a standard molar ratio of lipids of cholesterol:DSPC:SM-102:PLA-PEG of 38.5:10:50:1.5 and monitored in terms of encapsulation efficiency (EE%), Z-avg particle size (nm) and luciferase activity in HEK293 cells 72 hrs post transfection, respectively. The results are represented as the mean ± STD of three different samples. The molar ratio of lipids was cholesterol:DSPC:SM-102:PLA-PEG of 38.5:10:50:1.5 (DNA-LNP-F2).

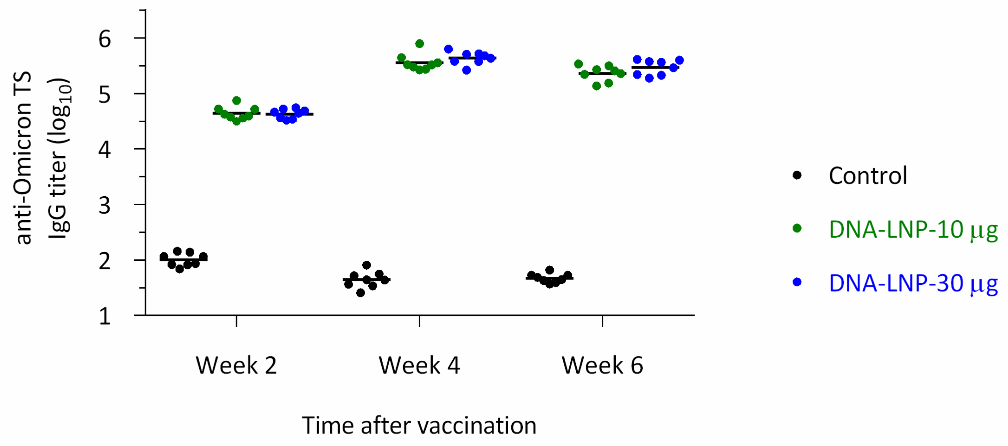

Figure S6. Omicron TS-specific IgG titers in hamsters injected with a two-dose TSomi DNA-LNP vaccination regimen

Hamsters (n = 8) were injected with TSomi DNA-LNP through the i.m. route on Day 0. At week 2, all hamsters were boosted through i.m. injection of the same vaccine formulations. The IgG antibodies in serum against trimeric spike protein are expressed as individual values with the GMT, which were obtained by enzyme-linked immunosorbent assay (ELISA).

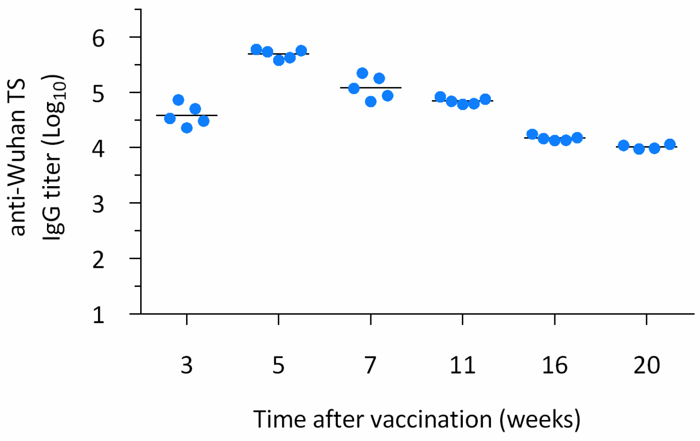

Figure S7. Wuhan TS-specific IgG titers in hamsters injected with a two-dose Wuhan mRNA-LNP vaccination regimen

Hamsters (*n* = 5) injected with 10 µg Wuhan mRNA-LNP through the i.m. injection on Day 0 and Day 21. The IgG antibodies in serum against trimeric spike protein are expressed as individual values with the GMT, which were obtained by enzyme-linked immunosorbent assay (ELISA).
